# Suspensão dos Diuréticos Tiazídicos na Doença Renal Crônica Avançada. Tempo de Rever um Antigo Conceito

**DOI:** 10.36660/abc.20230115

**Published:** 2023-10-03

**Authors:** Rodrigo Bezerra, Flávio Teles de Farias, Audes Feitosa, Wilson Nadruz, Andrea Araújo Brandão, Weimar Kunz Sebba Barroso

**Affiliations:** 1 Universidade Federal de Pernambuco Laboratório de Imunopatologia Keizo Asami Recife PE Brasil Universidade Federal de Pernambuco - Laboratório de Imunopatologia Keizo Asami, Recife, PE – Brasil; 2 Universidade Federal de Alagoas Maceió AL Brasil Universidade Federal de Alagoas, Maceió, AL – Brasil; 3 Universidade de Pernambuco Pronto Socorro Cardiológico de Pernambuco Recife PE Brasil Universidade de Pernambuco - Pronto Socorro Cardiológico de Pernambuco (PROCAPE), Recife, PE – Brasil; 4 Universidade Estadual de Campinas Campinas SP Brasil Universidade Estadual de Campinas (UNICAMP), Campinas, SP – Brasil; 5 Universidade do Estado do Rio de Janeiro Departamento de Doenças do Tórax Rio de Janeiro RJ Brasil Universidade do Estado do Rio de Janeiro - Departamento de Doenças do Tórax, Rio de Janeiro, RJ – Brasil; 6 Universidade Federal de Goiás Serviço de Cardiologia Liga de Hipertensão Arterial Goiânia GO Brasil Universidade Federal de Goiás - Serviço de Cardiologia, Liga de Hipertensão Arterial, Goiânia, GO – Brasil

**Keywords:** Insuficiência Renal Crônica/complicações, Diuréticos, Pressão Sanguínea, Hipertensão, Hipocalcemia

As Diretrizes Brasileiras de Hipertensão Arterial das Sociedade Brasileira de Cardiologia (SBC), Sociedade Brasileira de Hipertensão (SBH) e Sociedade Brasileira de Nefrologia (SBN) recomendam o uso de diuréticos de alça em substituição dos tiazídicos nos pacientes com doença renal crônica (DRC) estágios 4 e 5, ou seja, ritmo de filtração glomerular (RFG) ≤ 30ml/min/1,73m^2^.^1^ Esta orientação está alinhada com outras diretrizes, como por exemplo, a Diretriz Europeia de Hipertensão (ESC/ESH 2018) que afirma que os diuréticos tiazídicos e tiazídico-símile são menos efetivos em pacientes com RFG < 45ml/min/1.73m^2^e inefetivos quando o RFG < 30ml/min/1,73m^2^. ^2^ No entanto, o estudo que estabeleceu este conceito data de 1961 e avaliou apenas 11 pacientes, sendo que apenas 5 destes apresentavam RFG < 37ml/min/1,73m^2^. A despeito disso, este conceito gerou um dogma exportado para os livros textos, propagado por décadas como uma verdade incontestável.^3^

Após a publicação do estudo ALLHAT em 2002 e a demonstração do impacto positivo da clortalidona sobre o controle pressórico e desfechos cardiovasculares, os tiazídicos ganharam destaque no tratamento da hipertensão arterial (HA), sendo hoje considerados primeira linha de tratamento, seja em monoterapia ou em associação com outros fármacos.^4^ Além disso, a HA e a doença renal crônica (DRC) frequentemente coexistem, seja como causa ou consequência da perda de função renal e muitos dos estudos utilizados para nortear a produção das diretrizes de hipertensão arterial utilizaram um grande número de pacientes com DRC. Como exemplo, nos estudos SPRINT e ALLHAT, 28% e 23,7% dos pacientes respectivamente, apresentavam DRC estágios 3 e 4.^4,5^

**Figura 1:**
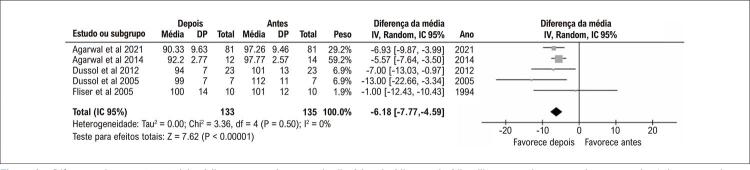
– Diferença da pressão arterial média antes e após o uso do diurético tiazídico ou tiazídico-like em paciente com doença renal crônica avançada.

Recentemente nosso grupo publicou uma *random-effects* metanálise com o intuito de avaliar a efetividade dos diuréticos tiazídicos e tiazídico-símile sobre o controle da HA em pacientes com RFG < 45ml/min/1,73m^2^ (DRC estágios 3b, 4 and 5). Nessa análise foram incluídos 5 ensaios clínicos com um total de 214 pacientes e RFG que variou de 13,0 ± 5,9ml/min/1,73m^2^ a 26,8 ± 8,8ml/min/1,73m^2^. Dentre os principais achados, foi observada uma redução significativa na pressão arterial (PA) média (Figura 1) que foi acompanhada de um aumento da fração de excreção de sódio e cloro e de uma redução no RFG, não sendo descritos episódios graves de injúria renal aguda.^6^ Dentre os estudos incluídos nesta metanálise, destaca-se o CLICK Trial, um estudo duplo-cego, randomizado, controlado por placebo em pacientes portadores de DRC estágio 4 (GFR médio no início do estudo de 23,2 ± 4,2ml/min/1,73m^2^) e PA acima da meta, com achados de redução significativa dos níveis pressóricos com a clortalidona.^7^

Além do controle da HA, o uso dos tiazídicos na DRC estágios 4 e 5 tem mais algumas vantagens. Uma delas é a tendência a um discreto aumento nos níveis de cálcio em consequência à redução da calciúria. Tal efeito se contrapõe à tendência a hipocalcemia que ocorre nos estágios mais avançados da DRC, pela deficiência de 25 (OH) vitamina D. Outra vantagem é seu efeito espoliador de potássio, que poderia ajudar na manutenção de drogas importantes como os bloqueadores do receptor de angiotensina (BRA) e inibidores da enzima conversora de angiotensina (IECA), muitas vezes suspensos por hipercalemia. Alinhado com esta hipótese, destaca-se que no CLICK trial 60,5% dos pacientes do grupo clortalidona faziam uso concomitante de diurético de alça. Neste sentido, pode-se especular que a adição de tiazídico a diurético de alça na população com DRC avançada poderia espoliar ainda mais o potássio e ter um efeito adjuvante na manutenção de drogas inibidoras do sistema renina angiotensina aldosterona.

Levando em consideração que a HA é a segunda causa de DRC no mundo e que 10% da população mundial têm algum grau de disfunção renal, o uso deste ponto de corte de 30ml/min/1.73m^2^ impede que milhares de pacientes se beneficiem dos já demonstrados efeitos protetores cardiovasculares dos diuréticos tiazídicos. Algumas diretrizes mais recentes como as do KDIGO Work Group já não concordam com a limitação do uso dos tiazídicos em pacientes com RFG > 30ml/min/1,73m^2^.^8^

Desta forma, baseado na produção de novas evidências científicas ao longo das últimas décadas, sugerimos que a recomendação ao uso apenas de diuréticos de alça ou mesmo de não utilizar os tiazídicos em pacientes com disfunção renal grave seja revista pelas sociedades científicas.
