# Supplementary material for: Suspensão dos Diuréticos Tiazídicos na Doença Renal Crônica Avançada. Tempo de Rever um Antigo Conceito
Source: Arq Bras Cardiol. 2023 Oct 3;120(9):e20230115. [Article in Portuguese] doi: 10.36660/abc.20230115 (PMC10547429; doi:10.36660/abc.20230115)
Supplement: Translation

# Translation: Suspension of Thiazide Diuretics in Advanced Chronic Kidney Disease. Time to Review an Old Concept

**Authors:** Rodrigo Bezerra, Flávio Teles de Farias, Audes Feitosa, Wilson Nadruz, Andrea Araújo Brandão, Weimar Kunz Sebba Barroso

**Affiliations:** 1 Brazil Universidade Federal de Pernambuco - Laboratório de Imunopatologia Keizo Asami, Recife, PE – Brazil; 2 Brazil Universidade Federal de Alagoas, Maceió, AL – Brazil; 3 Brazil Universidade de Pernambuco - Pronto Socorro Cardiológico de Pernambuco (PROCAPE), Recife, PE – Brazil; 4 Brazil Universidade Estadual de Campinas (UNICAMP), Campinas, SP – Brazil; 5 Brazil Universidade do Estado do Rio de Janeiro - Departamento de Doenças do Tórax, Rio de Janeiro, RJ – Brazil; 6 Brazil Universidade Federal de Goiás - Serviço de Cardiologia, Liga de Hipertensão Arterial, Goiânia, GO – Brazil

**Keywords:** Renal Insufficiency, Chronic/complications, Diuretics, Blood Pressure, Hypertension, Hypocalcemia

The Hypertension Guidelines of the *Sociedade Brasileira de Cardiologia* (SBC), Brazilian Society of Hypertension (BSH), and Brazilian Society of Nephrology (BSN) recommend the use of loop diuretics to replace thiazides in patients with chronic kidney disease (CKD) stages 4 and 5, who have glomerular filtration rate (GFR) ≤ 30ml/min/1.73m^2^.^1^

These guidelines are in line with other guidelines, such as the European Hypertension Guideline (ESC/ESH 2018), which states that thiazide and thiazide-like diuretics are less effective in patients with GFR < 45ml/min/1.73m^2^ and ineffective when GFR < 30ml/min/1.73m^2^.^2^ However, the study that established this concept dates from 1961 and evaluated only 11 patients, among whom 5 had GFR < 37ml/min/1.73m^2^. Despite this, this concept generated a dogma exported to textbooks, which has been propagated for decades as an incontestable truth.^3^

After the publication of the ALLHAT study in 2002 and the demonstration of the positive impact of chlorthalidone on blood pressure (BP) control and cardiovascular outcomes, thiazides gained prominence in the treatment of arterial hypertension (AH) and are now considered the first line of treatment, whether in monotherapy or association with other drugs.^4^ In addition, AH and CKD often coexist as a cause or consequence of loss of renal function, and many of the studies used to guide the elaboration of AH guidelines used a substantial number of patients with CKD. For example, in the SPRINT and ALLHAT studies, 28% and 23.7% of the patients, respectively, had CKD stages 3 and 4.^4,5^

Recently, our group published a random-effects meta-analysis to evaluate the effectiveness of thiazide and thiazide-like diuretics on controlling AH in patients with GFR < 45ml/min/1.73m^2^ (CKD stages 3b, 4, and 5). This analysis included five clinical trials with 214 patients and GFR ranging from 13.0 ± 5.9ml/min/1.73m^2^ to 26.8 ± 8.8ml/min/1.73m^2^. Among the main findings, a significant reduction in mean arterial BP was observed (Figure 1), followed by an increase in the excretion fraction of sodium and chlorine and a reduction in GFR, with no description of severe episodes of acute kidney injury.^6^ Among the studies included in this meta-analysis, the CLICK Trial stands out: a double-blind, randomized, placebo-controlled study including patients with stage 4 CKD (mean GFR at baseline of 23.2 ± 4.2ml/min/1.73m^2^) and BP above the target, which found a significant reduction in BP levels with chlorthalidone.^7^

**Figure 1:** – Difference in mean arterial pressure before and after the use of a thiazide or thiazide-like diuretic in a patient with advanced chronic kidney disease.

In addition to AH control, the use of thiazides in CKD stages 4 and 5 has some other advantages. One is the trend towards a slight increase in calcium levels due to the reduction in calciuria. This effect contrasts with the trend towards hypocalcemia that occurs in the more advanced stages of CKD due to 25(OH) vitamin D deficiency. Another advantage is its potassium-wasting effect, which could help maintain essential drugs such as angiotensin receptor blockers (ARB) and angiotensin-converting enzyme (ACE) inhibitors, often suspended due to hyperkalemia. In this sense, it can be speculated that adding a thiazide to a loop diuretic in the population with advanced CKD could further deplete potassium and have an adjuvant effect in the maintenance of drugs that inhibit the renin-angiotensin-aldosterone system. In line with this hypothesis, it should be noted that 60.5% of the patients in the chlorthalidone group used a loop diuretic concomitantly in the CLICK trial.

Considering that AH is the second cause of CKD in the world and that 10% of the world’s population has some degree of renal dysfunction, the use of this cutoff point of 30ml/min/1.73m^2^ prevents thousands of patients from benefiting from the acknowledged cardiovascular protective effects of thiazide diuretics. Some more recent guidelines, such as those of the KDIGO Work Group, no longer agree with limiting the use of thiazides in patients with GFR > 30ml/min/1.73m^2^.^8^

Thus, based on the production of new scientific evidence over the last few decades, we suggest that the recommendation to use only loop diuretics or even not to use thiazides in patients with severe renal dysfunction should be reviewed by scientific societies.
